# Effective Apical Infection of Differentiated Human Bronchial Epithelial Cells and Induction of Proinflammatory Chemokines by the Highly Pneumotropic Human Adenovirus Type 14p1

**DOI:** 10.1371/journal.pone.0131201

**Published:** 2015-07-13

**Authors:** Elena Lam, Mirja Ramke, Gregor Warnecke, Sonja Schrepfer, Verena Kopfnagel, Thomas Dobner, Albert Heim

**Affiliations:** 1 Institute of Virology, Hannover Medical School, Hannover, Germany; 2 Heinrich-Pette-Institute, Department Viral Transformation, Hamburg, Germany; 3 Department of Cardiothoracic, Transplantation and Vascular Surgery, Hannover Medical School, Hannover, Germany; 4 University Heart Center Hamburg, Transplant and Stem Cell Immunobiology Laboratory, Universitäts Klinikum Eppendorf, Hamburg, Germany; 5 Department of Dermatology, Hannover Medical School, Hannover, Germany; University of Regensburg, GERMANY

## Abstract

**Background:**

Only a few pneumotropic types of the human adenoviruses (e.g. type B14p1) cause severe lower respiratory tract infections like pneumonia and acute respiratory distress syndrome (ARDS) even in immunocompetent patients. By contrast, many other human adenovirus (HAdV) types (e.g. HAdV-C5) are associated mainly with upper respiratory tract infections. This is in accordance with a highly physiological cell culture system consisting of differentiated primary human bronchial epithelial cells which are little susceptible for apical HAdV-C5 infections.

**Objective and Methods:**

We hypothesized that a pneumotropic and highly pathogenic HAdV type infects differentiated human bronchial epithelial cells efficiently from the apical surface and also induces proinflammatory cytokines in order to establish ARDS and pneumonia. Therefore, the apical infection of differentiated primary human bronchial epithelial cells with the pneumotropic and virulent type HAdV-B14p1 was investigated in comparison to the less pneumotropic HAdV-C5 as a control.

**Results:**

Binding of HAdV-B14p1 to the apical surface of differentiated human bronchial epithelial cells and subsequent internalization of HAdV DNA was 10 fold higher (p<0.01) compared to the less-pneumotropic HAdV-C5 one hour after infection. Overall, the replication cycle of HAdV-B14p1 following apical infection and including apical release of infectious virus progeny was about 1000-fold more effective compared to the non-pneumotropic HAdV-C5 (p<0.001). HAdV-B14p1 infected cells expressed desmoglein 2 (DSG2), which has been described as potential receptor for HAdV-B14p1. Moreover, HAdV-B14p1 induced proinflammatory chemokines IP-10 and I-Tac as potential virulence factors. Interestingly, IP-10 has already been described as a marker for severe respiratory infections e.g. by influenza virus A H5N1.

**Conclusions:**

The efficient "apical to apical" replication cycle of HAdV-B14p1 can promote endobronchial dissemination of the infection from the upper to the lower respiratory tract. Simultaneous induction of proinflammatory cytokines probably contributes to the high virulence of HAdV-B14p1.

## Introduction

Only four types (type 4 of species HAdV-E, types 3, 7 and 14p1 of species HAdV-B) of the 71 human adenovirus (HAdV) types frequently cause lower respiratory tract infections, presenting as pneumonia and acute respiratory distress syndrome (ARDS).

HAdV-B14 was first described as respiratory pathogen in Dutch military recruits in the late 1950s [1] and found to be associated with pharyngoconjunctival fever in college students but was not associated with severe clinical diseases [2]. Subsequently, the significance of the other pneumotropic types HAdV-E4 and -B7 for severe lower respiratory tract infections (including ARDS) in military recruits was recognized in the 1960s and a vaccine for these types was developed [3].

The re-emerging HAdV-B14p1 was first isolated in the US, related to fatal pneumonia outbreaks [4] and predominated beginning from 2006 [5]. HAdV-B14p1 causes lower respiratory tract infections not only in military recruits (as HAdV-E4 and -B7) but also in the civilian population affecting infants, young adults, and elderly individuals with and without preexisting medical conditions [4]. These findings indicated a higher virulence of the re-emergent HAdV-B14p1 even compared to HAdV-E4 and HAdV-B7. Recently HAdV-B14p1 was also isolated in Canada, China, Ireland and Scotland from pneumonia patients [6–9].

So far, the organo-tropism and virulence factors of HAdV-B14p1 are not yet fully elucidated. Probably, all HAdV types can be transmitted by droplets and replicate in the upper respiratory tract. Efficient endobronchial (luminal) spread of the HAdV-B14p1 infection to the lower respiratory tract and induction of inflammatory cytokines may be essential for a rapid onset of pneumonia. Animal models to study HAdV pneumonia like the cotton rat [10] have drawbacks due to the species specificity of HAdV. Their replication in rodents is inefficient, expression of their late genes is incomplete [11] and the release of infectious virus progeny is aborted. Therefore, the application of high titer viral inoculums (e.g. 10^6^ to 10^10^ plaque forming units/ml) was required to establish a pneumonia phenotype in animal models [10].

Differentiated human bronchial epithelial cells, which were polarized and differentiated at the air-liquid interface, are a model to study apical HAdV infections of the bronchial tract [12]. Luminal (apical) HAdV-C5 infection of differentiated human bronchial epithelial cells proved to be inefficient compared to basal infection [12–14], as the primary receptor for HAdV-C5, the coxsackie and adenovirus receptor (CAR) is mainly expressed on the basolateral side. This may limit the luminal, endobronchial spread of the HAdV-C5 infection from the upper to the lower respiratory tract. In accordance with this finding, the few cases of HAdV-C5 pneumonias have been limited to immunocompromised patients and may be the result of lower respiratory tract infections by viremia [15].

In the present study, the in vitro model system of differentiated human bronchial epithelial cells was used for the first time to study HAdV-B14p1 infection. Apical HAdV-B14p1 infection was found to be more effective compared to HAdV-C5 infection and resulted in efficient apical release of infectious virus progeny. In vivo, this may promote the luminal (endobronchial) spread of HAdV-B14p1 from the upper to the lower respiratory tract. Furthermore, HAdV-B14p1 infection induced proinflammatory responses by inducing chemokines IP-10 and I-Tac. The attracting of T-cells and macrophages to the side of infection may result in a massive immune response leading to severe pneumonia and ARDS.

## Materials and Methods

### Cell culture model of differentiated human bronchial epithelial cells

**Fig 1:**
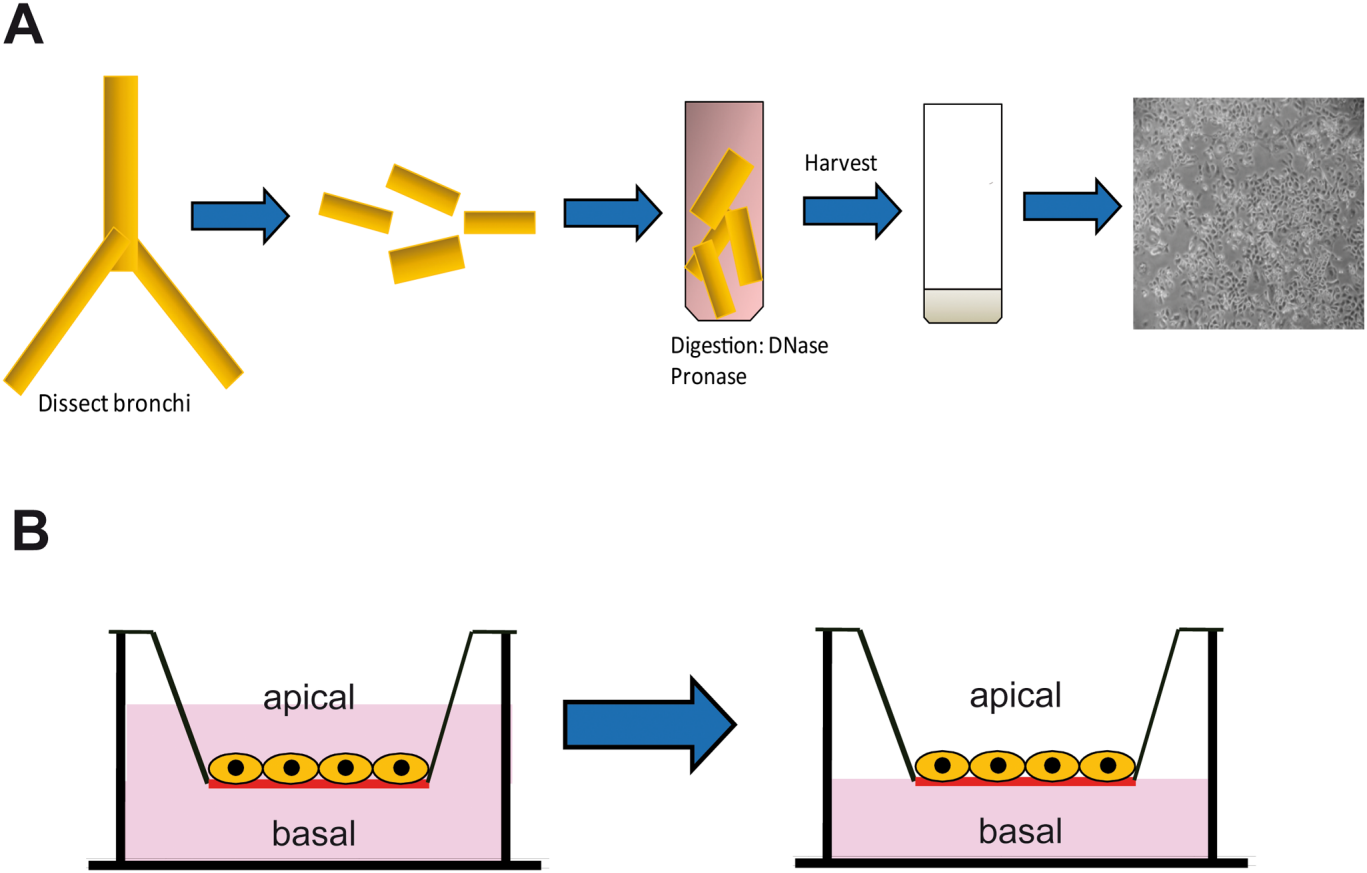
Schematic diagram of primary human bronchial epithelial cell isolation and cultivation. **(A)** Isolation procedure for primary human bronchial epithelial cells **(B)** Primary human bronchial epithelial cells cultured under submerged (left) and air-liquid interface conditions (right).

Primary human bronchial epithelial cells were isolated from lung explants of patients suffering from pulmonary fibrosis, pulmonary embolism and chronic obstructive pulmonary disease as described previously (Fig 1A) [16,17]. Written informed consent was provided by the tissue donors. The project was approved from the ethical review committees (Ethikkommission der Ärztekammer Hamburg, WF-011/13; Ethikkommssion der Medizinischen Hochschule Hannover 122–2007). Cells were seeded onto collagen I coated, semipermeable 0.33 cm^2^ polyester (PET) filter membrane inserts (0.4 μm pore size, Corning Costar, New York) for 24-well plates. Cells from a single donor were used for each experimental set (including HAdV-B14p1, -C5 and mock infection) in order to limit inter-donor differences. Experiments were repeated with bronchial epithelial cells derived of 4 different donors. Cells were seeded at a density of 10^5^ cells per insert. After cells had reached confluence (with about 5 x 10^5^ cells per insert), the airway medium was removed from the upper compartment and cells were cultured under air-liquid interface conditions (Fig 1B). Subsequently, the epithelial cells were cultured for 6 weeks for differentiation until differentiation was observed by expression of cilia (S1 Fig) and high transepithelial electrical resistance (TEER) >400 Ωcm^2^.

### Virus stocks

HAdV-C5 (ATCC VR-5) and a clinical isolate of HAdV-B14p1 (kindly provided by Michael Carr, National Virus Reference Laboratory, University College, Dublin, Ireland) [6] were propagated on A549 (ATCC CCL-185) cells and harvested with three freeze and thaw cycles at 70–80% cytopathic effect (CPE) for production of crude virus stocks. Ratio of particles/infectious units as measured by the tissue culture infectious dose 50% method (TCID_50_) was 4.35 for HAdV-C5 (TCID_50_/ml: 3.16 x 10^10^; particles/ml: 1.33 x 10^11^) and 19.7 for HAdV-B14p1 (TCID_50_/ml: 10^10^; particles/ml: 1.97 x 10^11^).

### Adenovirus infection

**Fig 2:**
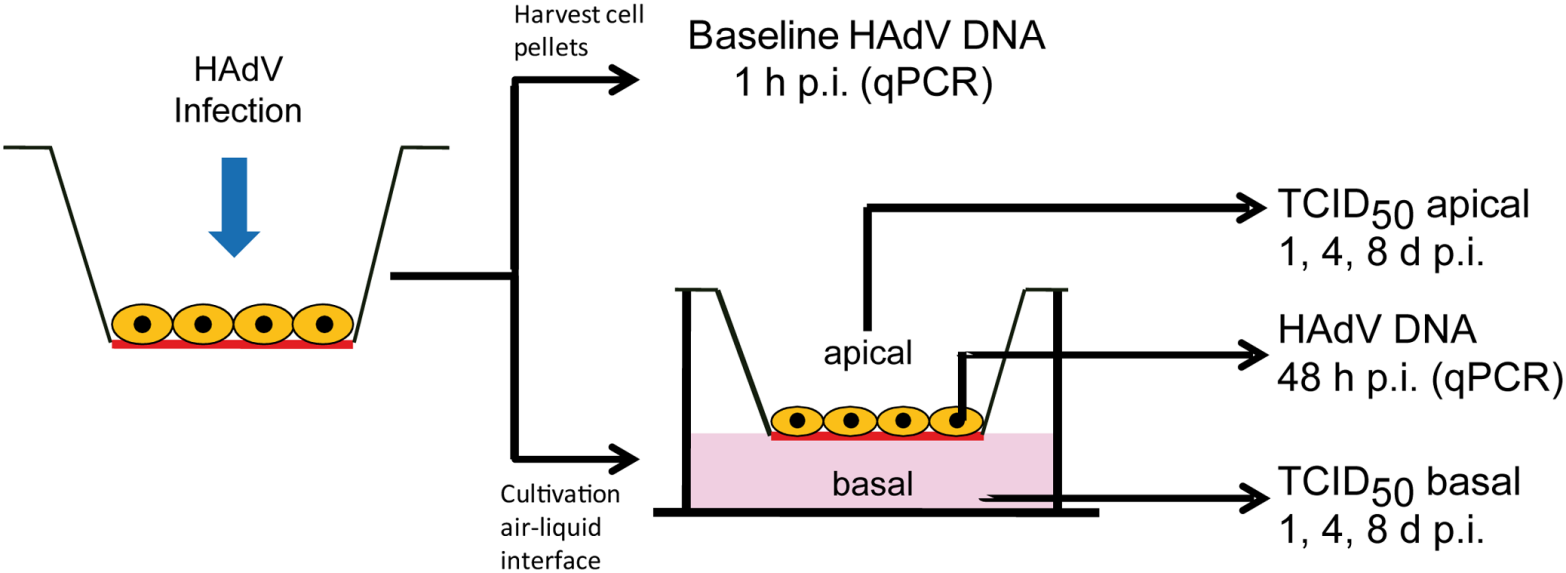
Schematic diagram of the infection and experimental procedure of differentiated human bronchial epithelial cells.

Quadruplicates of differentiated airway epithelial cells (5 x 10^5^ cells/ insert, size 0.33 cm^2^) were infected with HAdV on the apical surface at a multiplicity of infection (moi) of 10 TCID_50_/cell for 1 h at 37°C (Fig 2). On day 1, 4 and 8 post infection (p.i.) virus was collected by washing the apical surface with 100 μl DMEM^-^. These samples and medium samples from the lower compartment were titered for infectious virus progeny by the TCID_50_ technique on A549 cells.

### Quantitative HAdV PCR

Quantitative polymerase chain reaction (qPCR) was used to quantify viral genomes in the stock virus preparation and in infected cells and supernatants. Cells were sampled at 1 h and 48 h p.i. after unbound virus was removed by washing. DNA was extracted using the DNA Blood Kit (Qiagen, Hilden, Germany) according to the manufacturer´s protocol. Quantitative PCR was performed using the Platinum Quantitative PCR SuperMix-UDG (Invitrogen, Darmstadt, Germany) and specific forward (5´-GCCACGGTGGGGTTTCTAAACTT-3´, Adenoquant-1) and reverse (5´-GCCCCAGTGGTCTTACATGCACATC -3´, Adenoquant-2) primers and probe (FAM 5´-TGCACCAGACCCGGGCTCAGGTACTCCGA -3´ TAMRA) (Eurogentec, Seraign, Belgium) [18].

### Immunostaining and confocal microscopy

Differentiated cells were fixated on the PET membranes as described previously [19]. The PET membrane was kept attached to the insert during the whole staining and washing procedure. The primary antibody was applied as a 30 μl drop on a parafilm to the PET membrane and additionally 50 μl of the antibody was applied to the apical side of the cell layer. The washing steps were carried out by placing the insert into a 24 well plate and adding PBS to the upper and the lower compartment. Secondary antibodies were applied as the primary antibodies. Nuclei were counterstained with DAPI. After final washing the PET membrane was cut from the insert and mounted on a glass slide with the cells facing the cover slip using Mowiol (Sigma-Aldrich, St. Louis, MO) as mounting medium. Primary antibodies used were a polyclonal rabbit occludin (OCLN) antibody (1:40 diluted; Invitrogen, Paisley, UK), a monoclonal mouse CAR antibody (RmcB, 1:100 diluted, kindly provided by M. Bergelson), a monoclonal mouse desmoglein 2 (DSG2) antibody (Clone 6D8, 1:50 diluted, Santa Cruz Biotechnology, Santa Cruz, CA), and a FITC conjugated monoclonal adenovirus antibody (Millipore, Bilerica, MA). Secondary antibodies were anti rabbit conjugated with FITC and anti mouse conjugated with dsRed (1:200 diluted, Jackson, Immunoresearch, West Grove, PA). Images were acquired with a Leica DM IRB Laser Scanning Confocal Microscope.

### Microarray-based mRNA expression analysis

Samples for the “Whole Human Genome Oligo Microarray V2” (ID 026652, Agilent, Santa Clara, CA) were prepared and the microarray was processed as described in the “One-Color Microarray-Based Gene Expression Analysis Protocol V5.7” (Agilent). Scanning was conducted with the Agilent Micro Array Scanner G2565CA, data extraction was performed with the “Feature Extraction Software V10.7.3.1” (extraction protocol: GE1_107_Sep09.xml).

Processed signals of the green channel (“gPS”) were normalized by linear scaling: All gPS values of one sample were multiplied by a scaling factor calculated as: 1500 / 75th percentile of the respective array. All normalized gPS values that fell below an intensity border of 15 were substituted by the respective surrogate value of 15.

### Quantification of CXCL10 and CXCL11 chemokine expression

Proinflammatory chemokines CXCL10 (IP-10) and CXCL11 (I-Tac) protein levels were quantified by enzyme immunoassay (Quantikine Immunoassay CXCL10 and CXCL11, R&D systems, Minneapolis, MN).

## Results

### Apical HAdV infection of differentiated bronchial epithelial cells

**Fig 3:**
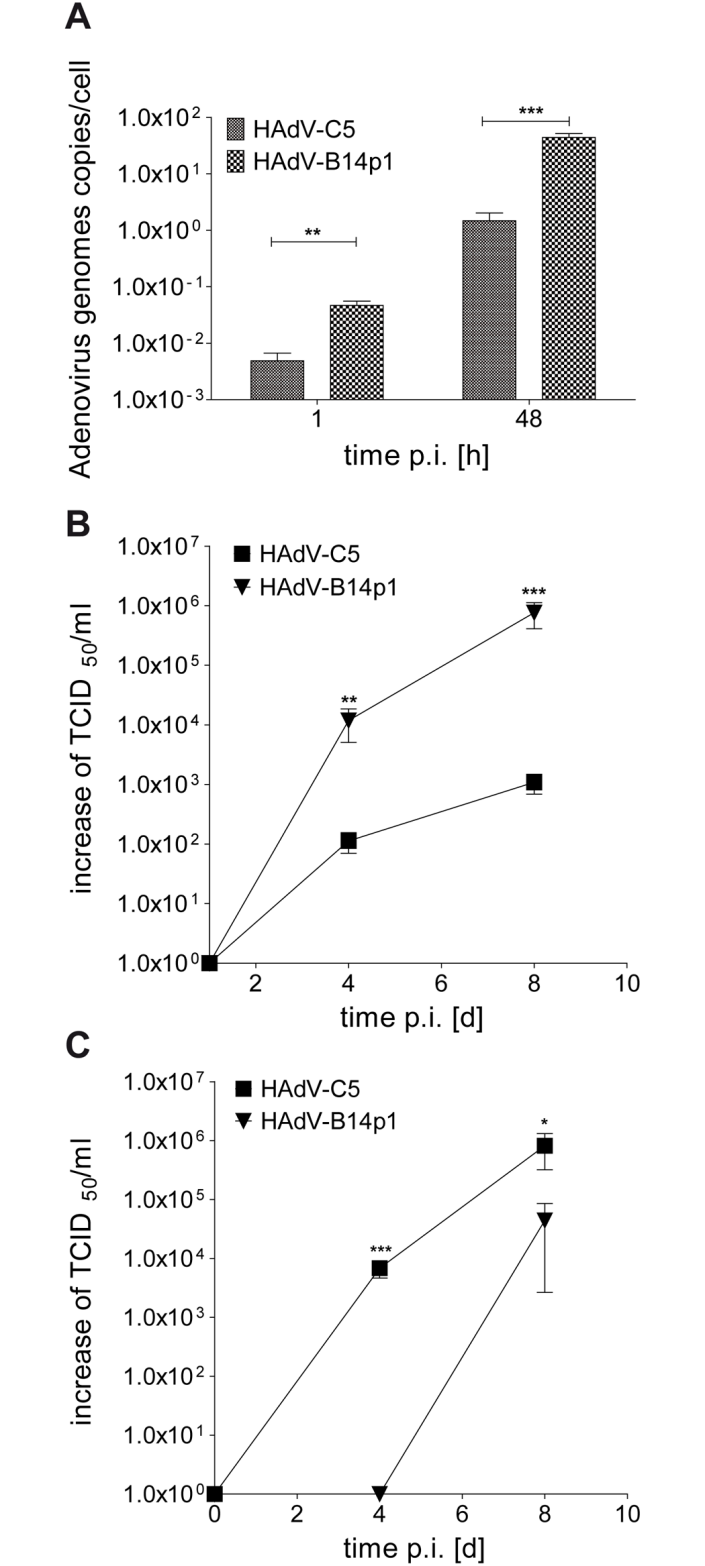
Efficient apical infection of differentiated human bronchial epithelial cells with HAdV-B14p1. **(A)** Intracellular HAdV genomes were quantified by qPCR at 1 h and 48 h p.i. (** p< 0.01; *** p< 0.001, unpaired t-test) **(B)** Release of infectious virus progeny at the apical side of the differentiated bronchial epithelial cell layer as determined by the TCID_50_ method on day 1, 4, and 8 p.i. (** p< 0.01; *** p< 0.001, unpaired t-test). **(C)** Release of infectious virus progeny at the basal side of the differentiated bronchial epithelial cell layer as determined by the TCID_50_ method on day 1, 4, and 8 p.i. (* p< 0.05; *** p< 0.001, unpaired t-test). The TCID_50_ values in B and C are normalized against the input virus titers measured on day 1 p.i. and set to 1 x 10^0^ because the TCID_50_ values measured on day 1 are probably remaining viral particles originating from the virus inoculum.

After apical infection of differentiated human bronchial epithelial cells, cell associated HAdV-B14p1 DNA concentrations were significantly higher (p<0.01) compared to HAdV-C5 DNA levels at 1 h p.i. This result indicated more efficient receptor binding and internalization of HAdV-B14p1 (Fig 3A). HAdV genome replication resulted in elevated levels of HAdV-B14p1 DNA compared to HAdV-C5 DNA at 48 h p.i. (Fig 3A).

### Apical and basal release of infectious virus progeny

The release of infectious HAdV progeny at the apical surface of differentiated human bronchial epithelial cells was monitored with help of the TCID_50_ method. Release of HAdV-B14p1 was significantly higher compared to HAdV-C5 on day 4 p.i. (p<0.01) and 8 d p.i. (p<0.001) (Fig 3B).

To compare directed basal to apical release after apical HAdV-C5 and HAdV-B14p1 infection, the infectious virus progeny release at the basal surface of differentiated human bronchial epithelial cells was additionally monitored. HAdV-C5 reached significant higher titers compared to HAdV-B14p1 on day 4 p.i. (p<0.001) and day 8 p.i. (p<0.05) (Fig 3C).

CPE was finally observed on day 12 p.i. and transepithelial resistance dropped (S2 Fig) as cells were lysed and tight junctions disrupted. An early and temporary decrease of TEER was only observed in HAdV-C5 infection but cells were morphologically unchanged and viable.

### Desmoglein 2 (DSG2) expression

**Fig 4:**
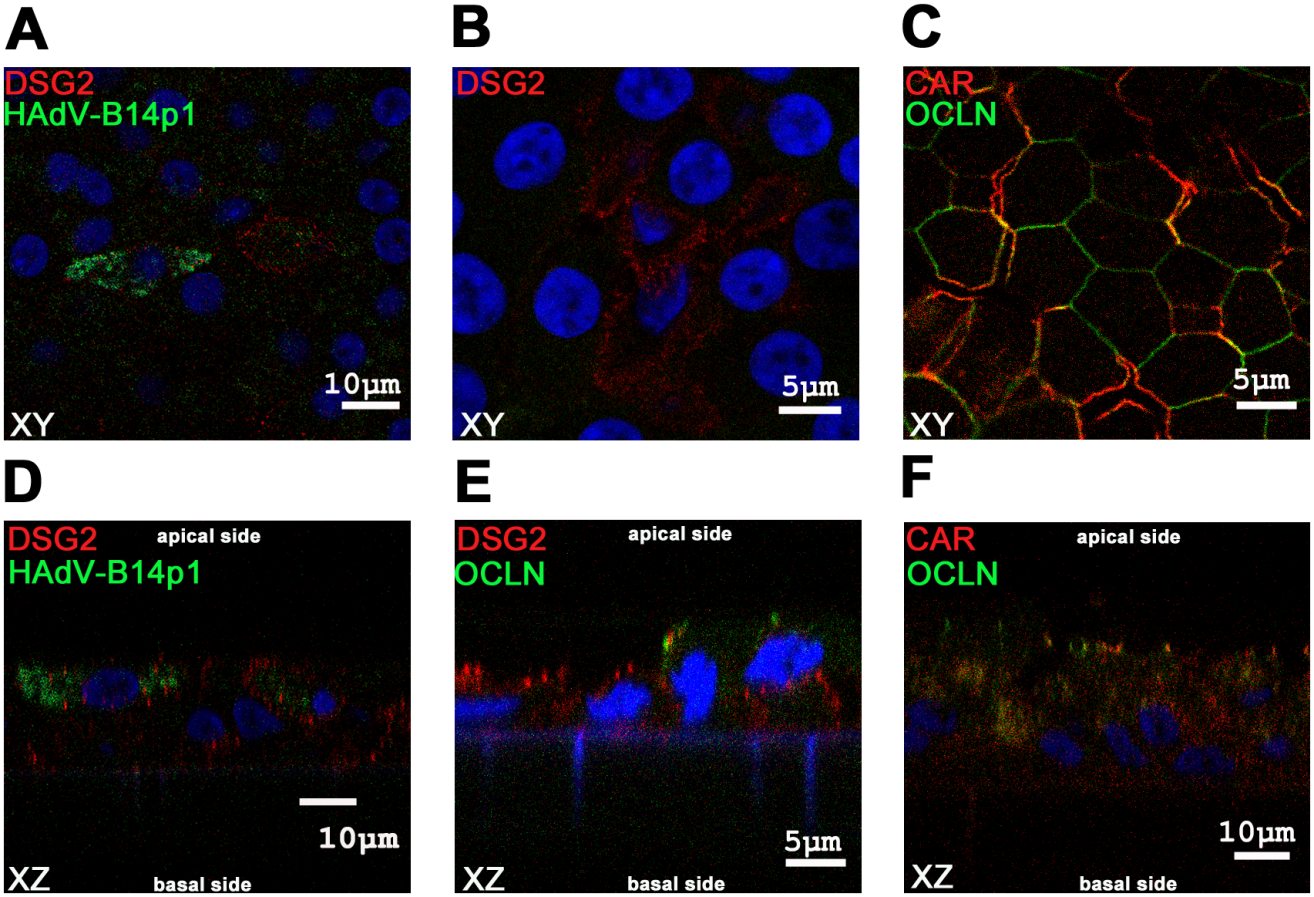
Immunofluorescence staining of differentiated human bronchial epithelial cells. Confocal microscopy immunofluorescence analysis of differentiated human bronchial epithelial cells. Figs A, B, C show XY planes, Figs D, E, F show XZ planes. **(A, D)** Cells were infected from the apical side with HAdV-B14p1 at a moi of 10 (TCID_50_/cell), fixated 4 days p.i. and stained for HAdV in green (FITC conjugated antibody) and the desmoglein 2 (DSG2 receptor) in red (dsRed antibody), the nucleus was counterstained in blue (DAPI). **(B, E)** Differentiated human bronchial epithelial cells stained for DSG2 receptor in red (dsRed) and occludin (OCLN) a tight junction marker in green (FITC), nucleus was counterstained in blue (DAPI). **(C, F)** Differentiated human bronchial epithelial cells were stained for CAR receptor in red (dsRed) and the tight junction marker OCLN in green (FITC) and the nucleus in blue (DAPI).

Bronchial epithelial cells were immunostained for HAdV hexon antigens and DSG2, the recently described novel adenoviral apical receptor [20], at day 4 post infection. DSG2 positive cells were found to be HAdV-B14p1 infected (Fig 4A–4D). Staining for HAdV-B14p1 was mainly cytoplasmic whereas DSG2 staining was mainly cell membrane associated. DSG2 was expressed close to the apical side of differentiated bronchial epithelial cells (Fig 4B–4E). For comparison, CAR, the main receptor for HAdV-C5, was expressed on the basolateral side and partially colocalized with the tight junction marker occludin (OCLN) (Fig 4C–4F).

### Induction of proinflammatory chemokines

**Fig 5:**
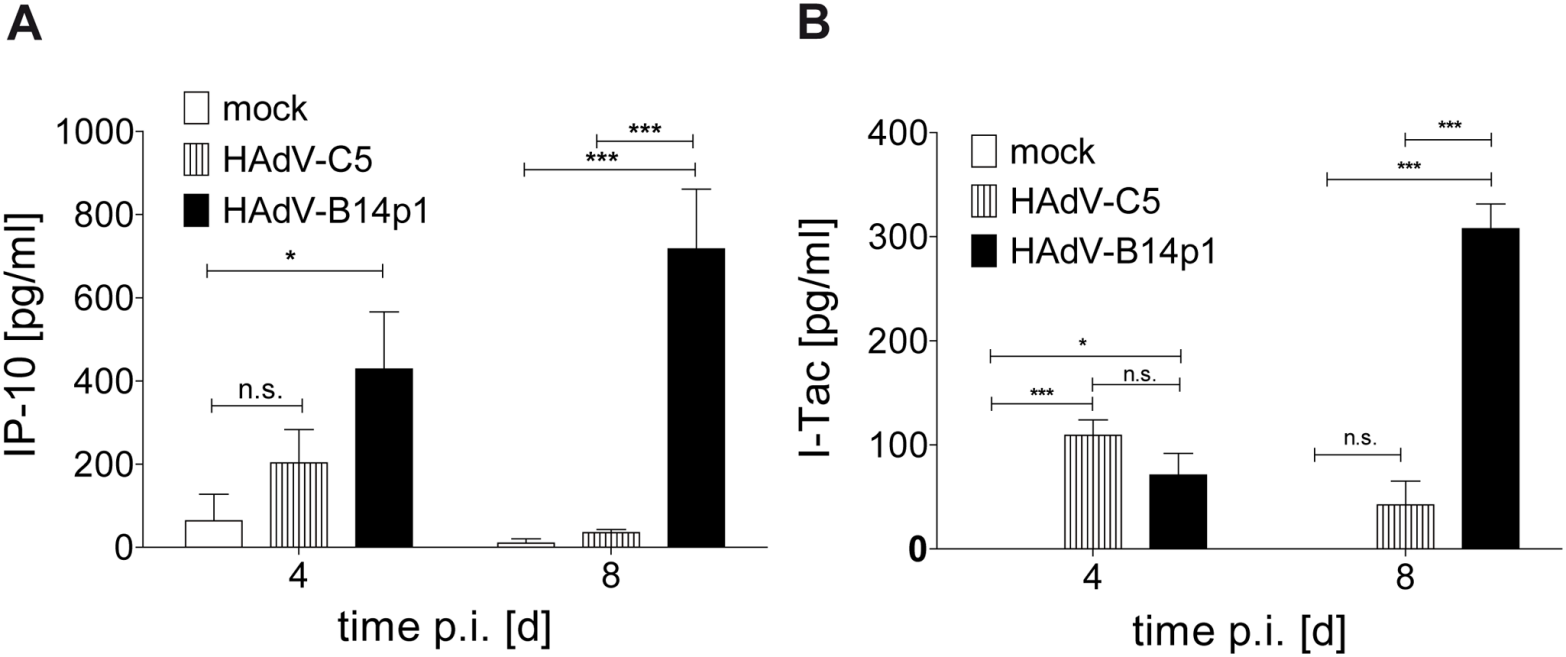
Induction of chemokines after apical HAdV infection of differentiated human bronchial epithelial cells. **(A)** IP-10 concentration in cell culture medium on day 4 and 8 p.i. as determined by ELISA **(B)** I-Tac concentration in cell culture medium on day 4 and 8 p.i. as determined by ELISA (n.s.: not significant, * p< 0.05; *** p< 0.001, two way ANOVA). Values shown are SEM values of quadruplicate infections.

The induction of the chemokine genes IP-10 (CXCL10) and I-Tac (CXCL11) and of the proinflammatory cytokine gene IL-6 was observed in a genome wide mRNA microarray analysis at 48 h post HAdV-B14p1 infection (S1 Table), but not in HAdV-C5 infected control cultures. Subsequently the release of chemokines IP-10 and I-Tac by HAdV-B14p1 by infected cell cultures was confirmed on the protein level by ELISA (Fig 5A and 5B).

## Discussion

Spread of HAdV infections from the upper respiratory tract, as the site of primary infection to the lower respiratory tract, seems to be a prerequisite for severe disease manifestations such as bronchiolitis, pneumonia and ARDS. For example, infection of the upper respiratory tract with the "classical" pneumotropic HAdV types 4 and 7 by droplets or smear infection can result in manifestation of ARDS [21–24]. By contrast vaccination with (non attenuated!) HAdV-E4 and -B7 in enteric-coated tablets is not associated with severe respiratory disease but induces protective immunity by enteric HAdV replication [25]. From the experience with this vaccine, we deduced the working hypothesis that efficient luminal spread form the upper to the lower respiratory tract is essential for onset of pneumonia. Similar experience with a vaccine is not available in case of the (re-)emerging pneumotropic type HAdV-B14p1 [4,5]. However, the endobronchial dissemination of HAdV-B14p1 was investigated in the present study due to its high clinical relevance. This endobronchial spread requires apical infection of differentiated human bronchial epithelial cells and apical release of infectious virus progeny as demonstrated for HAdV-B14p1 in the present study (Fig 3B). Binding of HAdV-B14p1 to the apical surface of bronchial epithelial cells and subsequent internalization was 10 fold higher compared to the less-pneumotropic HAdV-C5 (Fig 3A). This correlated well with the expression of DSG2, the recently described receptor for HAdV-B14p1 at the distal end of intercellular junctions [20]. Thus DSG2 should be accessible from the apical side, probably facilitating entry by opening intercellular junctions, whereas most other non pneumotropic HAdV types (including HAdV-C5) bind to CAR [26]. This may limit the infection efficiency of HAdV-C5 from the endobronchial (apical) side because only a low abundance splice variant of CAR has been observed on the apical surface of human bronchial epithelial cells [19].

Although not every cell positive for DSG2 was infected with HAdV-B14p1 in our model system, all infected cells were indeed located at the apical surface, indicating apical binding and entry of HAdV-B14p1 (Fig 4D). In addition, a few bronchial epithelial cells negative for DSG2 were also found to be HAdV-B14p1 infected (data not shown). This finding may be explained with down regulation of DSG2 expression in HAdV-B14p1 infected cells. On the other hand, initial infection of the differentiated pseudostratified layer may be facilitated by DSG2 but not limited to DSG2 positive cells suggesting the relevance of other cellular receptors for HAdV-B14p1. DSG2 expression has been described on polarized BT474, T84 and CaCo-2 cells [20]. In the present study DSG2 expression was detected for the first time on differentiated primary bronchial epithelial cells. However, the direct binding of HAdV-14p1 to DSG2 was not studied, thus the receptor usage of HAdV-14p1 on bronchial epithelial cells needs to be confirmed in a future study.

Other species HAdV-B types, like HAdV-B3 or -B7 were not included in present study, as these frequently isolated respiratory pathogens cause pneumonia only in a small subset of cases. Moreover, reports on the receptor usage of HADV-B3 are partially contradictory reporting the binding to CD46 [27] or CD80/86 [28] as their main receptor. Probably different HAdV-B3 strains could use different receptors which may be related to their different virulence and pneumotropism.

The temporary drop of the TEER (S2 Fig) observed on day 1–3 p.i. in HAdV-C5 infection might be explained by a faint "early CPE" due to free capsid proteins in the virus stocks [29]. An early CPE is caused mainly by the penton base protein [30]. Additionally, HAdV-C fiber proteins, produced in excess during virus replication, are able to disrupt tight junctions, allowing HAdV-C progeny to escape to the apical surface of a differentiated bronchial epithelium by a paracellular pathway [31]. This previous study already reported the efficient primary release of HAdV-C2 on the basolateral side, similar to our results with HAdV-C5 (Fig 3C). The overall (apical plus basal) release of infectious virus progeny was not significantly different between HAdV-C5 and HAdV-B14p1 when surveyed on day 4 and 8 p.i. More efficient genome replication and secondary cell to cell spread may have minimized the differences between HAdV-B14p1 and HAdV-C5 binding and entry. In spite of these effects, the apical to apical replication cycle of the highly virulent HAdV-B14p1 was far more effective and resulted in an about 1000-fold higher virus titer compared to HAdV-C5 infection (day 8 p.i., p<0.001) (Fig 3B).

The induction of proinflammatory and chemotactic cytokines may also be essential for severe inflammation of the lower respiratory tract. Induction of CXCL10 (IP-10) and IL-8 by an NF-kB pathway has been observed as a response of the cell to adenoviral infections [32]. In case of the pneumotropic type HAdV-B7, the induction of IP-10 was observed in type I and type II alveolar epithelial cells whereas induction of IL-8 was only observed in type I alveolar epithelial cells [33]. IL-8 induction seems to be inconsistent between HAdV types and the infected tissues or cells [33–35].

Interestingly, a clinical study found elevated IP-10 levels to be a potential biomarker for severe acute respiratory virus infections [36]. Human airway epithelial cells have already been shown to release IP-10 in response to influenza A H5N1 infection [37]. This is in congruence with the results of this study since HAdV-B14p1 infection of differentiated bronchial epithelial cells resulted in a significant IP-10 and additionally I-Tac induction and release. The induction of these chemokines by HAdV-B14p1 infection, may result in the infiltration of the infected lung with macrophages, activated T cells and NK cells and subsequently result in the expression of multiple proinflammatory cytokines as observed in ARDS [38,39].

In conclusion, the “apical to apical” replication cycle of pneumotropic HAdV-B14p1 could promote the endobronchial (luminal) spread of HAdV-B14p1 to the lower respiratory tract. Subsequent induction of proinflammatory cytokines by HAdV-B14p1 may lead to severe pneumonia and ARDS.

## Supporting Information

S1 Fig Scanning electron microscopy depicting of cilia on the apical surface of differentiated bronchial epithelial cells cultured for 6 weeks on the air—liquid interface. (TIF)

S2 Fig TEER of differentiated human bronchial epithelial cells during HAdV infection. TEER values were measured on differentiated human bronchial epithelial cells after HAdV infection from day 1 to day 15 p.i. An initial drop in resistance (day 1–3 p.i.) observed with HAdV-C5 infection might be due to a slight, reversible early CPE caused by the virus inoculum.
(TIF)

S1 Table Microarray data: All genes found to be upregulated (threshold value 2.0) in differentiated human bronchial epithelial cells by HAdV-B14p1 or HAdV-C5 infection compared to mock infection. Multiple listing of gene names (for example, see CXCL10 (IP-10), CXCL11 (I-TAC) and IL-6) indicated that the upregulation was detected by multiple, different probes. Genes were listed by relative signal intensity in HAdV-B14p1 infection vs. mock infection.
(XLS)
