# Supplementary figures and images for: Effective Apical Infection of Differentiated Human Bronchial Epithelial Cells and Induction of Proinflammatory Chemokines by the Highly Pneumotropic Human Adenovirus Type 14p1
Source: PLoS One. 2015 Jul 13;10(7):e0131201. doi: 10.1371/journal.pone.0131201 (PMC4500402; doi:10.1371/journal.pone.0131201)

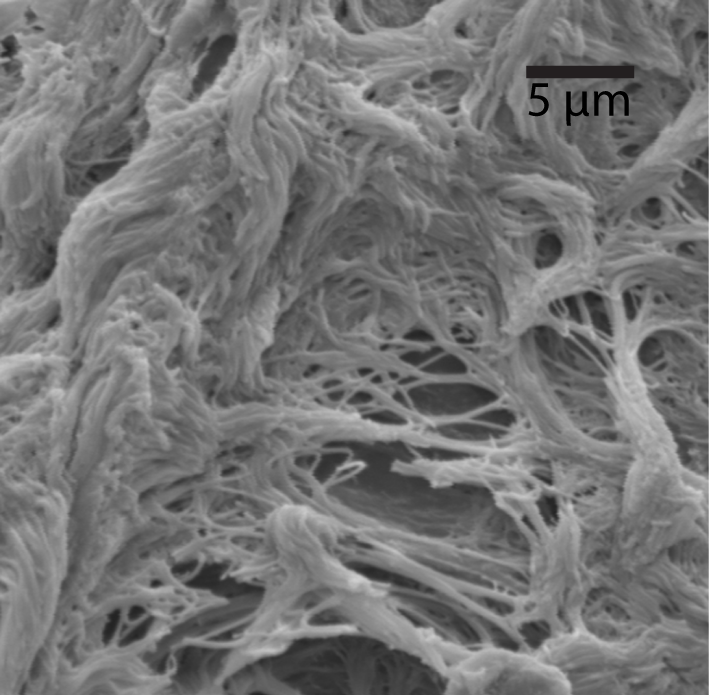

Supplement: S1 Fig — (TIF) [file pone.0131201.s001.tif]

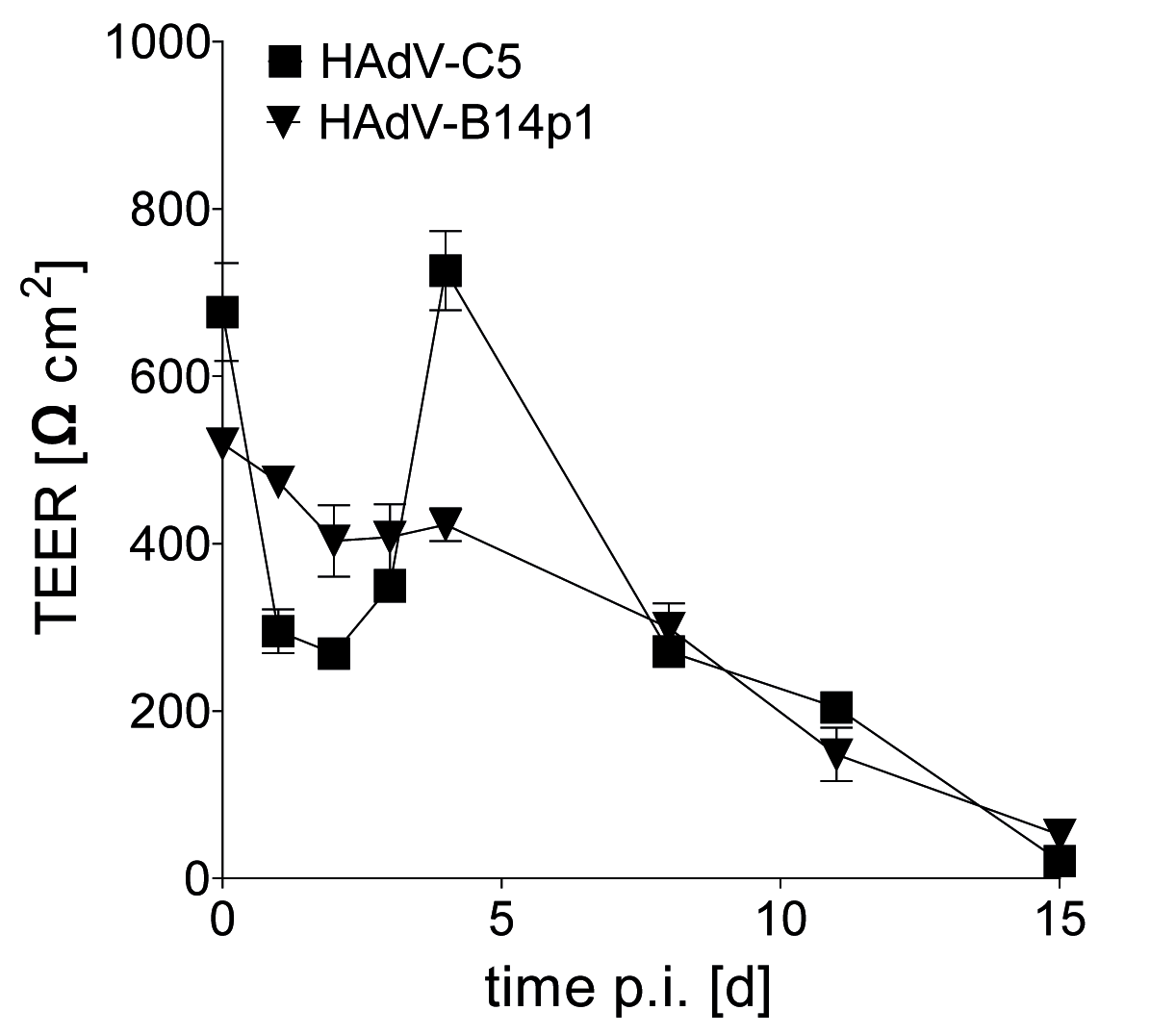

Supplement: S2 Fig — TEER values were measured on differentiated human bronchial epithelial cells after HAdV infection from day 1 to day 15 p.i. An initial drop in resistance (day 1–3 p.i.) observed with HAdV-C5 infection might be due to a slight, reversible early CPE caused by the virus inoculum. (TIF) [file pone.0131201.s002.tif]
